# Dynamic blood flow phantom for in vivo liquid biopsy standardization

**DOI:** 10.1038/s41598-020-80487-8

**Published:** 2021-01-13

**Authors:** Anastasiia Kozlova, Daniil Bratashov, Oleg Grishin, Arkadii Abdurashitov, Ekaterina Prikhozhdenko, Roman Verkhovskii, Natalia Shushunova, Evgeny Shashkov, Vladimir P. Zharov, Olga Inozemtseva

**Affiliations:** 1 Saratov State University, Saratov, Russia; 2 Skolkovo Institute of Science and Technology, Skolkovo Innovation Center, Moscow, Russia; 3 Prokhorov General Physics Institute of RAS, Moscow, Russia; 4 University of Arkansas for Medical Sciences, Little Rock, AR USA

**Keywords:** Melanoma, Bioinspired materials, Imaging and sensing

## Abstract

In vivo liquid biopsy, especially using the photoacoustic (PA) method, demonstrated high clinical potential for early diagnosis of deadly diseases such as cancer, infections, and cardiovascular disorders through the detection of rare circulating tumor cells (CTCs), bacteria, and clots in the blood background. However, little progress has been made in terms of standardization of these techniques, which is crucial to validate their high sensitivity, accuracy, and reproducibility. In the present study, we addressed this important demand by introducing a dynamic blood vessel phantom with flowing mimic normal and abnormal cells. The light transparent silica microspheres were used as white blood cells and platelets phantoms, while hollow polymeric capsules, filled with hemoglobin and melanin, reproduced red blood cells and melanoma CTCs, respectively. These phantoms were successfully used for calibration of the PA flow cytometry platform with high-speed signal processing. The results suggest that these dynamic cell flow phantoms with appropriate biochemical, optical, thermal, and acoustic properties can be promising for the establishment of standardization tool for calibration of PA, fluorescent, Raman, and other detection methods of in vivo flow cytometry and liquid biopsy.

## Introduction

Early disease diagnosis is significantly limited by the absence of methods for high sensitivity detection of extremely low-level quantity of disease-related markers, such as circulating tumor cells (CTCs), pathogenic bacteria, viruses, clots, and sickle cells. Many methods have been explored for this purpose including fluorescence, Raman, and photoacoustic (PA) spectroscopy and microscopy techniques^1–3^. PA imaging demonstrated significant benefits, such as deep penetration depth (up to 3–5 cm) to biological tissue^4^, safety, and noninvasiveness^1,5–10^. However, PA imaging is relatively slow, which prevents the study of dynamic events. Among different PA techniques, PA flow cytometry (PAFC) demonstrated the ability to assess large volumes of patient’s blood non-invasively in vivo*,* thus, allowing to detect ultralow concentrations of fast-moving pathological cells^11^. PAFC diagnosis platform has recently demonstrated significant clinical potential for detection of rare CTCs^11^, infections (for example, malaria^12^), exosomes^13^, bacteria, and blood clots (also called thrombi)^5,14,15^. Specifically, clinical potential of PAFC was demonstrated through detection of unprecedentedly low CTC concentration in melanoma patients directly in the bloodstream at the level of 1 CTC in 1 L of blood, which is 1000-fold sensitivity threshold improvement^11^.

As a new diagnostic tool, the PAFC platform requires specialized standardization and calibration procedures using a phantom with appropriate optical (absorption, scattering), thermal (heat conductivity), acoustic (speed of sound, acoustic attenuation), and PA contrast properties to establish standardized PA metrics. Comparison of the PA platforms for microscopy, spectroscopy, cytometry, or tomography is challenging due to differences in schemes, modes, data processing and analysis. The signals during in vivo measurements vary from animal to animal or from patient to patient depending on the vessel's depth and size, flow rate, or skin pigmentation. For ex vivo measurements the blood properties after sample collection are changing quickly within a few hours, therefore, it is problematic to have stable blood parameters during a long time. Altogether, this makes it difficult to use blood for quantitative accurate calibration of PA techniques ex vivo. Thus, there is an urgent need to develop new PA-specific standardization and calibration strategy. One of the key components of the calibration procedure is to have biologically-adequate vessel phantoms with flowing mimic normal and abnormal cells.

Different phantoms have been developed so far to calibrate PA techniques using various materials for biotissue properties’ modeling, such as agar, bovine gelatin, polyvinyl alcohol, and silica^16,17^, as well as transparent polymeric tubes^18^. The majority of blood phantoms, used for PA and ultrasound (US) methods, are still static or contain slowly moving (less than 0.1 cm/s) objects^19^, which is not appropriate for dynamic PAFC platforms with fast-moving cells with the velocity of up to 20–50 cm/s, as in human vessels, and the lifespan of objects in the irradiation point, which is less than 1 ms. Typically, variable dilutions of blood or different water/milk suspensions containing the determined amount of solid compounds, like polystyrene spheres or magnetic beads, are used as the flowing blood model^14,20–22^. Magnetic beads with varying size were used as models of red blood cells (RBC) or CTC and RBC aggregates, and transparent silica beads as white blood cells (WBC) model^22^. However, absorption spectra of typical PAFC targets, such as hemoglobin in RBCs or melanin in melanoma CTCs, are completely different from those of magnetic particles. Moreover, such magnetic beads are much heavier, which leads to their sedimentation and uneven flow of particles. This disrupts accurate calibration of PA instrument parameters.

In this work, we introduce the novel medically-relevant dynamic blood cell flow phantom, which has both blood-like background and circulating, rarely encountered, dispersed objects with variable concentration. The spectral properties, size distribution, and rheological behavior of the phantoms are similar to blood with rare CTCs, which allows us to calibrate and regularly evaluate the PA device parameters during routine clinical application.

## Results

**Figure 1:**
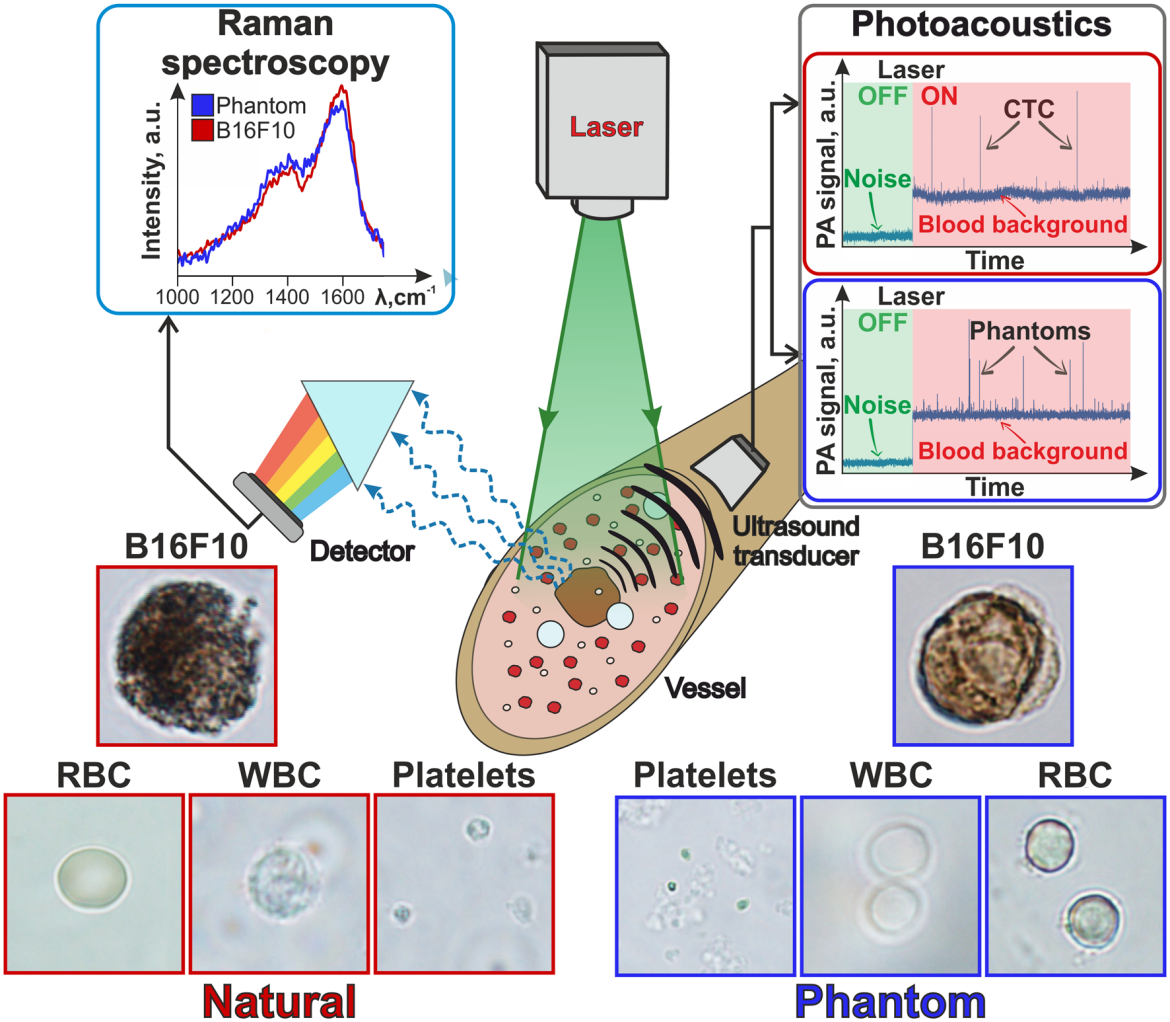
Principal scheme of in vivo flow cytometry. Left bottom—optical images of natural blood cells (red squares); right bottom—optical images of blood cell phantoms (blue squares); center—scheme of PA and Raman measurements (1064 nm laser for PA and 532 nm for Raman measurements); left top—Raman spectra of phantoms with melanin (blue) and B16F10 cells (red); right top—PA traces of blood with CTCs (red rectangle) and blood with phantoms (blue rectangle).

The purpose of the study was to develop and test the dynamic phantom, fully resembling the properties of the whole flowing blood and CTCs, rarely observed in cancer patients’ blood. We fabricated the system including model objects based on silica and Layer-by-Layer (LbL) assembled capsules incorporating hemoglobin molecules and natural melanin micro- and nanoparticles. The main challenge was to make them not only similar to real objects by their size and form, but also to simulate their optical properties and, partially, chemical compounds to ensure the ability to detect them using various analysis methods, such as Raman and PAFC (Fig. 1).

The key principle of PAFC has been described earlier^23–25^. In brief, periodic short laser pulses illuminate the absorbing targets, circulating in the blood, and the acoustic waves (referred to as PA signals), caused by laser heating, are detected by US transducer in real-time mode. Thus, PAFC provides a combination of high spectral specificity, due to various absorbance levels in different objects (for example, melanoma cells if compared with RBCs and WBCs), high spatial resolution in scattering media, and significant penetration depth typical of US techniques. This combination allows us to non-invasively detect markers associated with various diseases in the patient’s deep tissues and blood in vivo.

When passing through laser-irradiated volume, both natural melanoma cells (established cell line) and melanoma phantoms were shown to form similar positive PA peaks above the blood background due to higher localized absorption, than seen in RBCs (Fig. 1). Thus, the RBCs (5–6 µm) provided relatively strong PA blood background signals associated with hemoglobin-specific absorption spectra. Melanoma cells and their phantoms can be detected by the Raman spectra of melanin molecules as well, showing the same melanin peaks on the obtained spectra.

**Figure 2:**
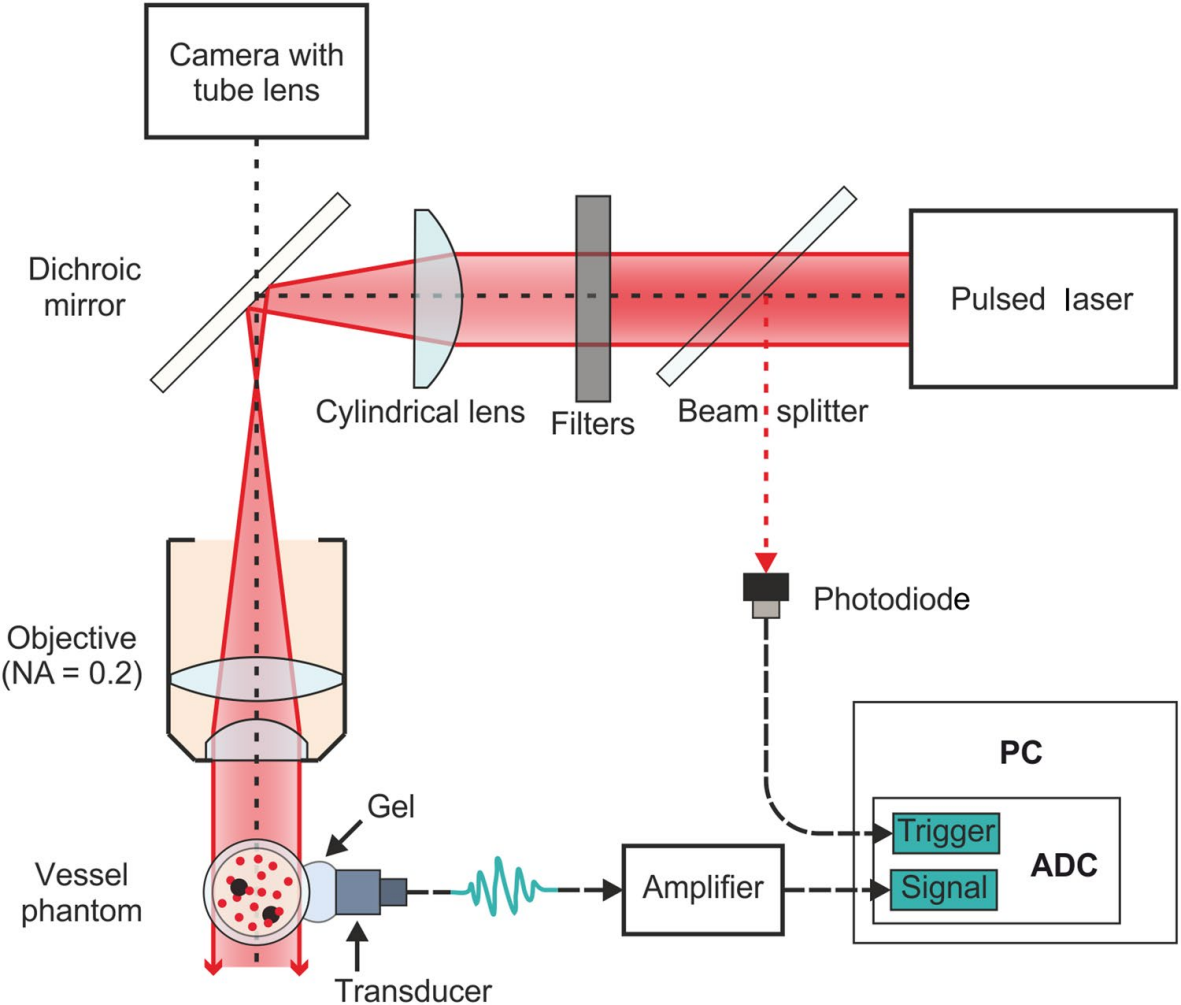
Scheme of PAFC setup. *ADC *analog-to-digital converter, *PC* personal computer. Vessel phantom is the plastic tube with flowing blood cell and CTC (black spots) phantoms. The filter indicates neutral filter (or several filters) for controlled laser energy attenuation. The transducer means US transducer for detection of laser-induced acoustic waves.

The PAFC setup was used to compare the PA signals from natural cells and their phantoms (Fig. 2, Fig. S1). Cylindrical lens and microscopic objective provide the laser beam focusing into the linear light sheet on the polydimethylsiloxane (PDMS) tube oriented perpendicularly in the direction of flowing phantoms. Neutral density filters were used to adjust laser energy reaching the tube. The US transducer was attached to the wall of the tube with the ultrasound gel to record laser-induced acoustic waves from the cell phantoms.

### Blood cell phantoms

**Figure 3:**
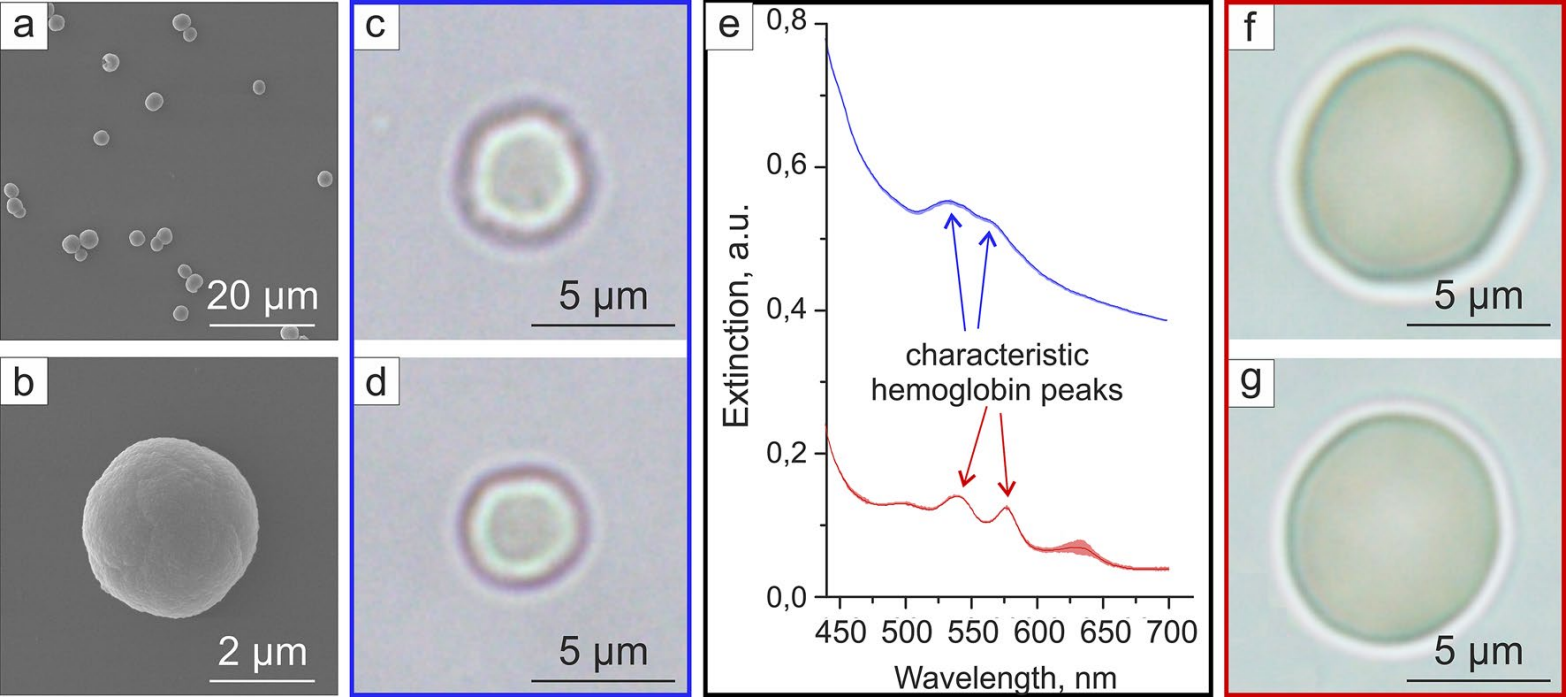
RBCs and their phantoms. (**a**,**b**) SEM images of RBC phantoms of different scales; (**c**,**d**) typical optical images of two RBC phantoms; (**e**) absorption spectrum of RBC (red) and RBC phantoms (blue), lighter background represents standard deviation; (**f**,**g**) typical optical images of two natural RBCs.

The RBCs were modeled by silica microparticles containing hemoglobin. The chemical precipitation method^26,27^ was used to create hemoglobin-contained calcium carbonate spherical microparticles with subsequent shell formation and dissolution of the cores. The average diameter of obtained hollow silica RBC phantoms was around 3.07 ± 0.76 µm (Fig. 3a–d). The optical images of RBC phantoms (Fig. 3c,d) demonstrated their morphological similarity to the natural RBCs (Fig. 3f,g). The difference in average extinction spectra intensity from phantoms and real RBCs was caused by the higher light scattering in phantom suspension due to the phantoms’ silica shells. Since the detector aperture was relatively low, only a part of scattered light was registered by the detector and caused this difference. A similar effect of increase in average absorption level of hemoglobin-containing particles comparing to hemoglobin was shown in^28^ and was caused by the nature of composite inorganic matrix. Nevertheless, the phantom suspension spectrum possessed the two peaks at 540 and 570 nm wavelength (Fig. 3e) characteristic of hemoglobin absorption in RBCs, which contributes to further PA signal generation and should provide a more prominent background within that wavelength range similar to real hemoglobin-contained cells.

**Figure 4:**
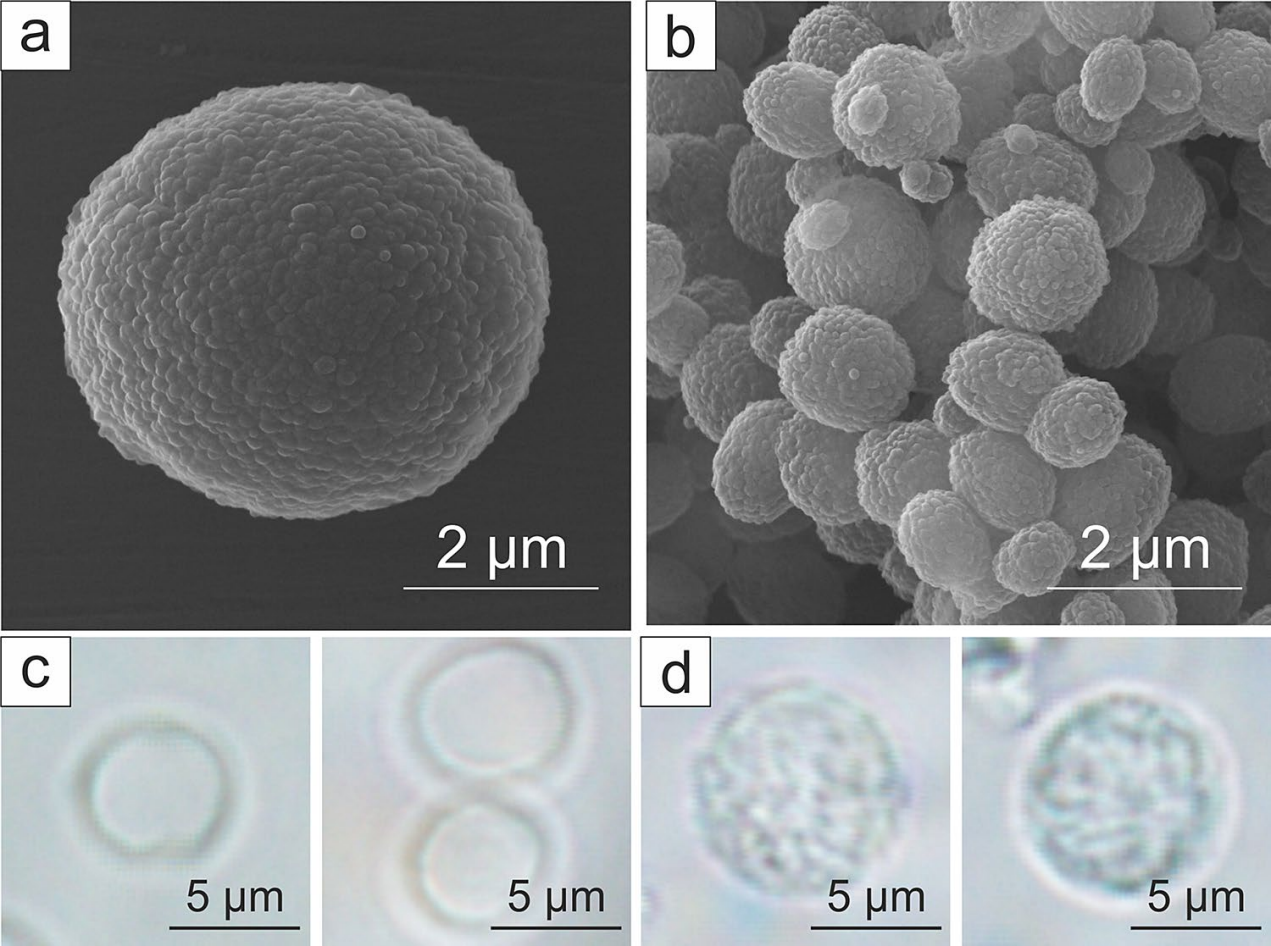
WBCs and platelets phantoms. (**a**,**b**) SEM images of (**a**) WBC phantoms and (**b**) platelets phantoms, (**c**) optical microscopy images of WBC phantoms, (**d**) optical microscopy images of human WBCs.

WBCs do not have any specific absorption peaks in the extended visible range^29^ and possess low absorption throughout it, therefore, we have chosen hollow silica spheres as their model. WBCs (Fig. 4d) and platelets were simulated by hollow, transparent, silica spheres with the diameter similar to corresponding blood cells: 5.4 ± 0.6 µm for WBC phantoms (Fig. 4a,c) and 1.1 ± 0.3 µm for platelet phantoms (Fig. 4b). Artificial (Fig. 4c) and natural (Fig. 4d) WBCs have the same round shape and transparency, nevertheless both RBC and WBC phantoms are slightly smaller than their real counterparts. It can make spectral PA response of these phantoms slightly different if compared with the real blood cells. However, the size of the obtained phantoms is limited by the possibility to produce larger spherical vaterite particles, especially in the presence of hemoglobin solution in the synthesis.

### Melanoma cells and melanoma cell phantoms

**Figure 5:**
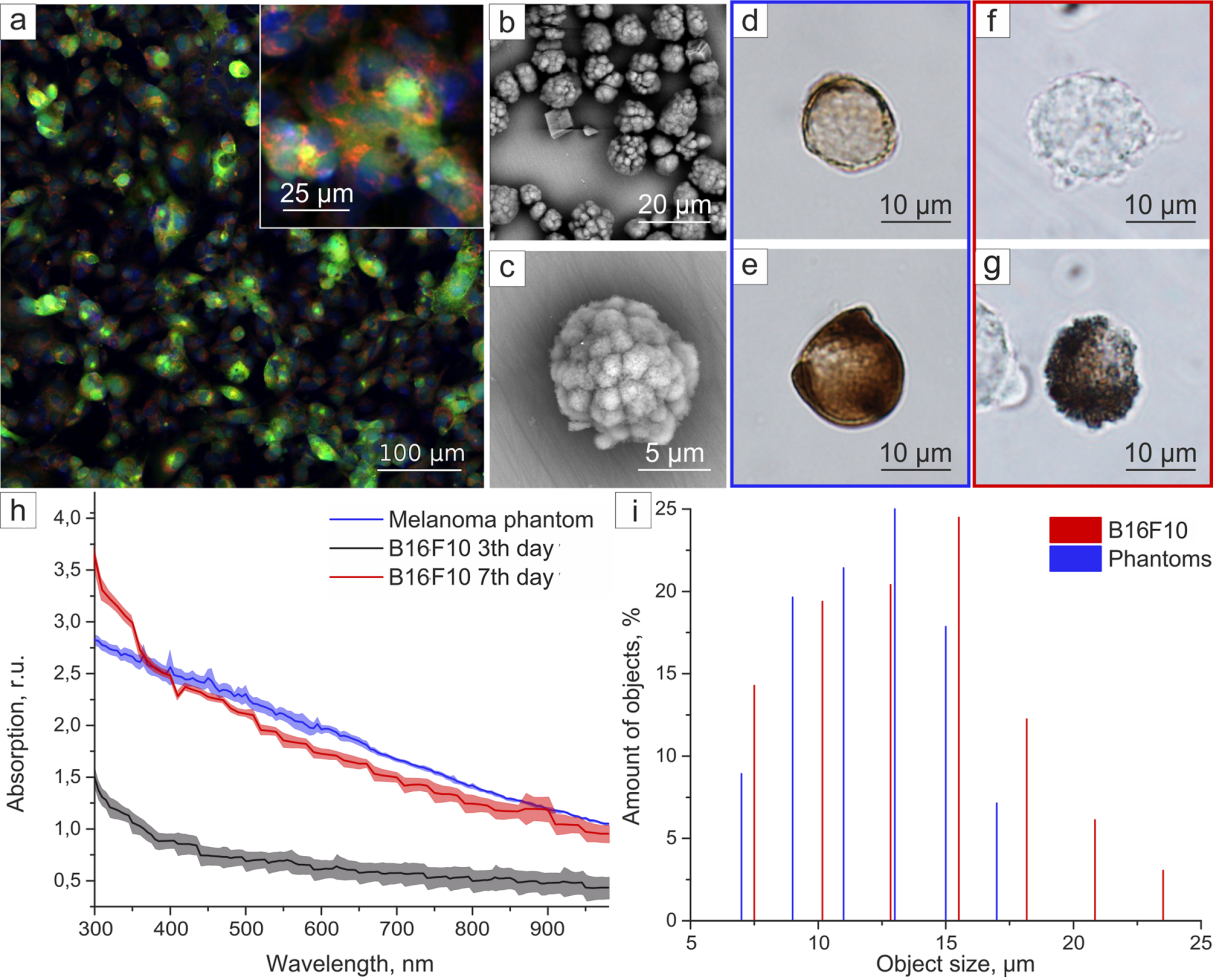
Murine melanoma cells and melanoma cell phantoms characterization. (**a**) confocal laser scanning microscopy (CLSM) image of B16F10 adherent cells stained with Calcein AM (green), Mitotracker (red) and DAPI (blue); (**b**) SEM image of CaCO_3_ templates; (**d**–**g**) optical microscopy images of (**d**,**e**) CTC phantoms and (**f**,**g**) B16F10 murine melanoma cells; (**h**) absorption spectra with standard deviation of phantom suspension (blue), B16F10 suspension after 3 (black), and 7 (red) days of cultivation (all spectra are normalized to absorption of dispersant, lighter background represents standard deviation); (**i**) size distribution histogram.

Melanoma cells circulating in the blood of patients are pigmented like melanocytes, due to melanin production that provides their easy in vivo PA detection. Typically, murine melanoma cell culture B16F10 is used as a model object in the melanoma study. B16F10 has a spindle, polyhedral shape on the growth substrate (Fig. 5a), but after the detachment from the tissue or any other surface it takes spherical shape (Fig. S3, Supplementary), enters the circulating blood and remains unchanged until extravasation^30^. The average diameter of the detached B16F10 cells is 13.4 ± 4.34 µm (Fig. 5i).

Melanoma CTC phantoms’ fabrication requires spherical templates with an average diameter close to that of B16F10^31^ and with similar optical properties, namely the absorption spectrum. Calcium carbonate particles were chosen as templates for phantom synthesis due to flexibility of obtainable parameters depending on synthesis conditions^32–38^. Typically, calcium carbonate synthesis results in amorphous CaCO_3_ particles of vaterite polymorphs slowly recrystallizing into cubic calcite^32,33^. By varying synthesis conditions and with the addition of surfactants, one can obtain various crystallite forms: aragonite^34^, needle-shaped^35^, flower-shaped^36,37^, and even raspberry-shaped^38^. For biomedical applications, biocompatibility and safety are essential factors, which limit available synthesis methods. For example, if the phantoms are used in experiments with laboratory animals, reagents like CTAB^34^ should not be used in this case. However, the size of calcium carbonate can vary gradually from 1 µm flower-shaped^39^ to nearly 50 µm with surfactant stabilization^37^.

The most suitable configuration for melanoma cells phantom is the raspberry-shaped calcium carbonate particles due to their size, shape, and high porosity^26^. Nevertheless, this form is a very challenging to synthesize due to its instability and a tendency to crystallize irregularly throughout the sample^38^. In the current study we managed to obtain raspberry-shaped calcium carbonate templates with less than 1% of calcite objects (see Fig. 5b,c). The templates that we produced were loaded with melanin, next the polymer shell was made using the method of sequential adsorption of polyelectrolytes. Further dissolution of sacrificial templates results in obtaining polymer capsules with melanin inside.

The real B16F10 cells are not homogeneous in terms of melanin content: some cells look dark on the optical microscopy images (Fig. 5g), due to high melanin concentration, but the majority of cells is almost transparent (Fig. 5f and Fig. S3, Supplementary). The fabricated melanoma phantoms have a 12 ± 3 µm average diameter (Fig. 5i) and simulate the non-homogeneous melanin content (Fig. 5d,e). Moreover, we found that the amount of melanin in cells increased along with cell growth under the proposed conditions (see “Methods”). To illustrate melanin content dependence on cultivation time, absorption of cell suspension in Dulbecco’s phosphate-buffered saline (DPBS) (10^7^ cells per mL) was measured after 3 and 7 days of cultivation (Fig. 5h). The absorption spectrum of phantom aqueous suspension was similar to cells on the 7th day of cultivation; it possessed the same slope and average absorption levels in volume (Fig. 5h).

We evaluated B16F10 and phantoms’ suspension spectra similarity by cosine distance method^40^. Each spectrum was presented as an n-dimensional vector where *n* is the number of wavelengths measured. Cosine value was calculated according to Eq. (1).

1 $$\cos \alpha = \frac{{{\varvec{a}} \cdot {\varvec{b}}}}{{\left| {\varvec{a}} \right| \cdot \left| {\varvec{b}} \right|}},$$

where ***a***—phantoms suspension absorption spectrum represented as a multidimensional vector, ***b***—B16F10 suspension absorption spectrum represented as a multidimensional vector, |***a***|—length of vector ***a***, |***b***|—length of vector ***b***, $\alpha$—angle between vectors. Resulting cosine was 0.9938 ± 0.0002, which confirmed the similarity of their optical properties with a 0.61% ± 0.02% difference.

### Photoacoustic and Raman measurements

A dynamic PA phantom was made from PDMS tube to model the blood vessel in the tissue with intrinsic scattering properties. The fabricated melanoma CTC, WBC, and platelet phantoms described above were used for the following PA measurements. Human RBCs were modeled by obtained RBC phantoms or hemoglobin solution. The 90 mg/mL concentration of pure hemoglobin solution was selected to match the PA signal level from natural blood.

The resulting phantom for the blood flow with rare CTCs in it provides constant PA background due to hemoglobin adsorption in a large number of RBC-like objects in the irradiated volume. Rare peaks appear when the highly adsorbing melanin-containing objects pass through it. The width of the peak depends on the flow speed and sometimes on the lifetime of melanin-containing particles destructed by the high-power laser beam.

PA signals were measured with pulsed laser excitation at a wavelength of 1064 nm, pulse duration of 2 ns, and different pulse energies. The signals were obtained from the flow of absorbing objects’ suspension, which was moved by the syringe pump at a constant speed.

**Figure 6:**
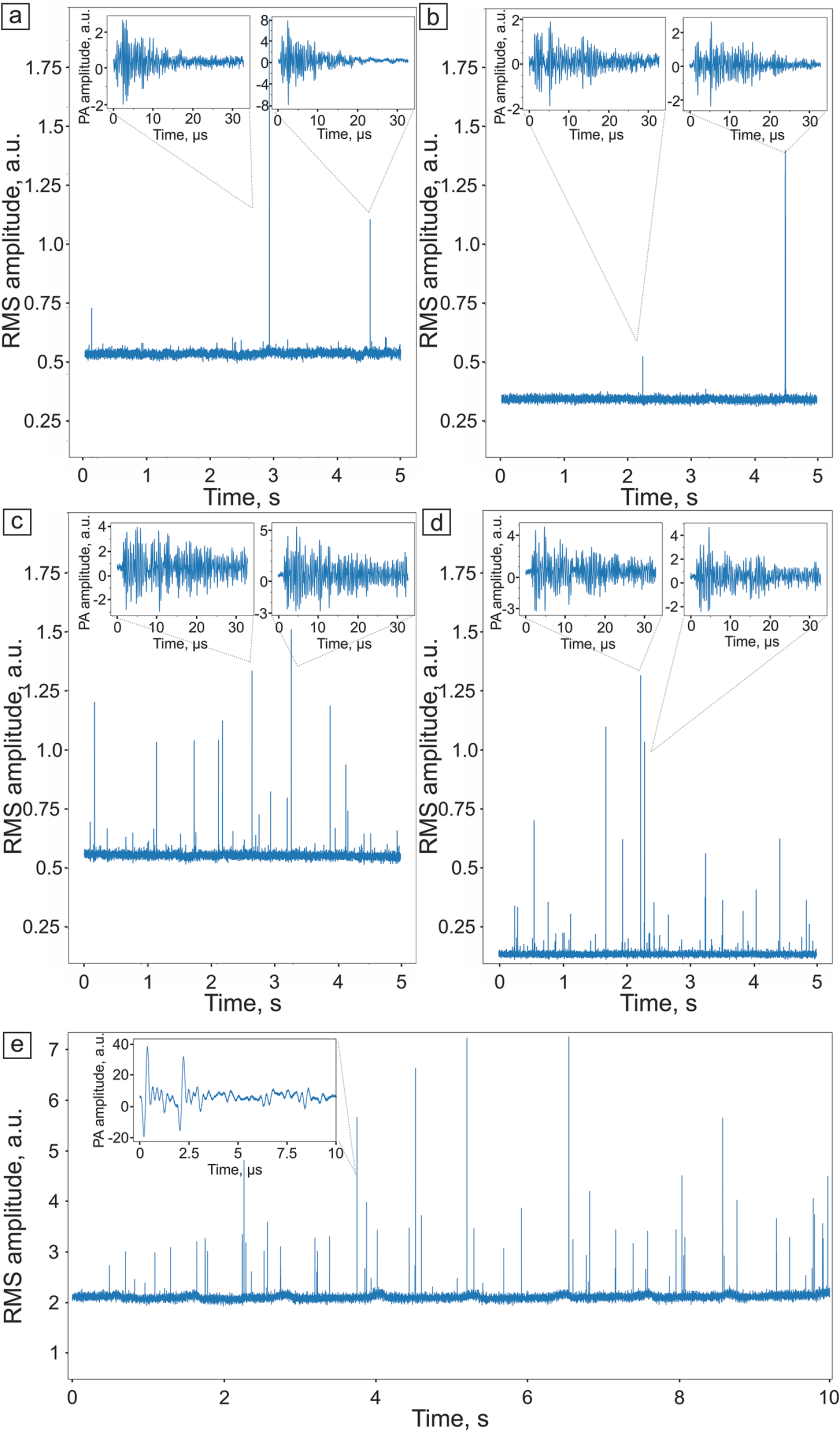
PA traces with signal waveform examples. (**a**) Human blood containing B1610 melanoma cells, (**b**) human blood containing melanoma cell phantoms, (**c**) hemoglobin solution containing melanoma cell phantoms, and (**d**) RBC phantoms’ suspension containing melanoma cell phantoms. Laser pulse energy, 100 µJ. (**e**) Mouse femoral vessel containing B16F10 in vivo. The insets show the examples of single waveforms.

The waveforms of PA signals from B16F10 targets (Fig. 6a) and phantoms with RBCs background (Fig. 6b), as well as melanin-containing phantoms in hemoglobin solution (Fig. 6c) or RBC phantoms background (Fig. 6d) were recorded by analog-to-digital converter (ADC) board. The pyroelectric signal generated in the PA sensor by the scattered laser light was removed from the waveform by providing a 1 µs delay between the laser pulse and the beginning of waveform acquisition. Every single waveform registered by the ADC board was cleared from high-frequency noise by the low-pass filter. It was provided by the convolution of the signal with the Gaussian function (with the cut-off frequency equal to maximum detection frequency of US sensor). The waveform was gated to the time range of PA signals by simple cut.

The two main parameters were evaluated from the registered PA signals to transform signal waveforms to PA traces (where each waveform was represented as a single time point in a PA trace). The first parameter was similar to that described in previous works, i.e. the peak-to-peak amplitude, which is the difference between the highest and the lowest amplitude of a signal within the gated time range. The second one was the root mean square (RMS) sum of differences between the signal and the mean amplitude in the gated time range corresponding to PA signals. It is proportional to the overall power of the PA signal and less sensitive to single point noise.

Sharp PA peaks on PA traces correspond to the passing individual strongly absorbing objects in the flow. The signal level between such outliers has Poisson-like distribution with relatively low variance. It represents the averaged background provided mostly by the hemoglobin of the RBCs and to some extent by the walls of the vessel itself. RBC phantoms’ concentration in the suspension was chosen to provide slightly fluctuating stable PA signal background (Fig. 6d and Fig. S4, Supplementary). The rate of PA signals from melanoma cells in the blood in vivo strongly depends on the patient, the disease stage, as well as cell pigmentation. Therefore, these signals are rarely observed on traces (Fig. 6a) and cannot provide constant control. However, the ability to change melanoma cell phantoms’ concentration in the sample from several objects per mL to several thousands allows us to solve this problem and manually adjust their concentration for the required purposes. Thus, we can make a sample with a large number of melanoma phantoms for the PAFC system calibration (Fig. 6c,d) and the low number of phantoms to model the real patient’s blood (Fig. 6b).

**Figure 7:**
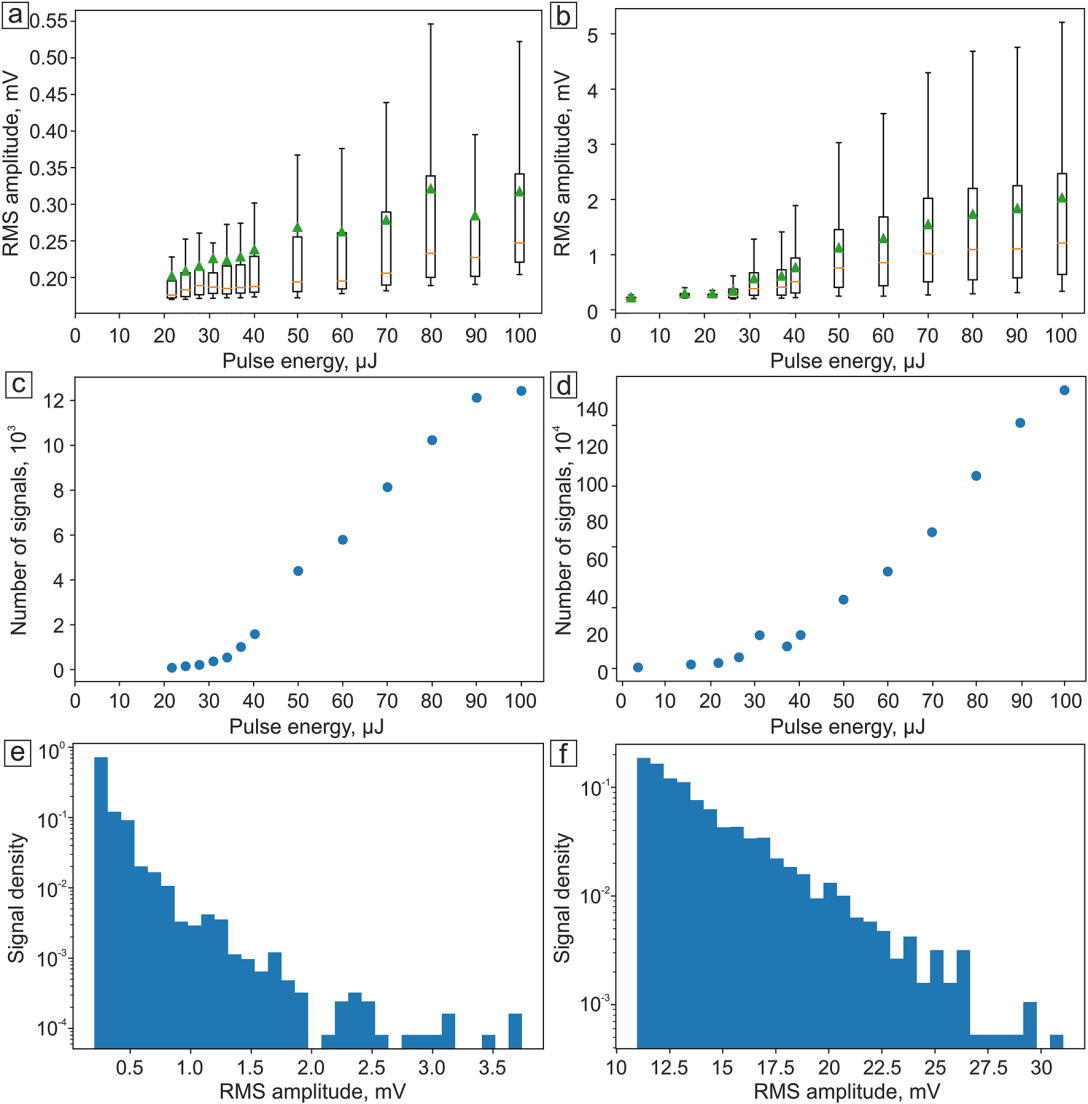
RMS amplitude of PA signal dependence on laser pulse energy for (**a**) B16F10 cells and (**b**) melanoma cell phantoms; dependence of number of signals on laser pulse energy for (**c**) B16F10 and (**d**) melanoma cell phantoms; signal density of (**e**) B16F10 and (**f**) melanoma cell phantoms for 50 µJ pulse energy. Orange lines on graphs (**a**), (**b**) represent median values, while green triangles reflect the mean values.

The signal amplitudes above the threshold from high absorbing targets were statistically evaluated (Fig. 7). The threshold was calculated for each trace by the addition of 3 standard deviations to the mean value of background that has Poisson-like statistical properties. The dependence of PA signal amplitudes’ parameters on the pulse energy shows clear threshold behavior. The minimum, maximum, mean, and median values of the amplitude do not surpass the threshold at low pulse energies and then slowly increase after 20–30 µJ (Fig. 7a,b). The number of signals above the threshold grows linearly proportional to the pulse energy (Fig. 7c,d). The histogram of signal amplitudes above the threshold (Fig. 7e,f) shows the distribution’s high-skewed character decreasing to the high values.

The resulting PA nonlinear signal behavior is associated with the formation of nano- and micro-bubbles induced by laser heating of melanin particles. The concentration of melanin clusters in both B16F10 cells and melanin-contained phantoms is non-uniform. Thus, by increasing the laser pulse energy the number of melanin clusters producing bubbles of boiling liquid also increases^41^. In the video S1 (Supplementary), one can see how the nanobubbles appear and boil near the absorbing objects in suspension when objects pass through the laser beam.

The histogram of amplitudes generated from different objects at the same laser power for both melanoma cells (Fig. 7e) and phantoms (Fig. 7f) demonstrates a strange downward character, resulting in a skewed statistical distribution. It is represented by the overlapping of two different distributions: linear PA signals from the flow cell and liquid flow and highly-skewed exponential PA signals from highly-absorbing objects in the suspension. The first distribution is non-skewed and similar to normal, however, its approximation by Gaussian leaves a part beyond 6 standard deviations. The second distribution describes signals from melanoma cells (or phantoms) in suspension. Melanin particles inside cells overheat, destroy, change their aggregative state and make surrounding liquid boil, forming gas–vapor bubbles. However, the particles could be just partially destructed by laser, which causes highly-skewed signal amplitude histogram (Fig. 7e,f), which declines from the background signal level to the random rare high values. When all the particles in the suspension become overheated, PA signal amplitude (Fig. 7a,b) and the number of signals (Fig. 7c) reach the plateau. This distribution is far from the normal, bell-shaped one, which makes it difficult to calculate typical statistical characteristics, such as the average, the median, etc. However, if we keep the parameters of PA signal detection the same (focus, flow cell position, etc.), the signal will remain constant, i.e. will not depend on the external influences. The same counting and analysis methods, as well as the same parameters for threshold and liner distribution assessment allows us to obtain typical statistical characteristics, required for further analysis.

We have also performed an additional PA measurement of B16F10 cells in mouse’s blood flow in vivo. The cell suspension in DPBS was injected into the mouse’s carotid artery, and the PA signals were detected on a femoral artery. The resulting PA trace (Fig. 6e) has sharp PA peaks differing in RMS amplitude, as were for the in vitro PA signals from B16F10 cells. However, some of the PA signals in vivo possessed higher amplitude, which could be caused by an additional aggregation of B16F10 and RBCs in the mouse’s blood flow. The waveforms of PA signals in vivo had two main peaks instead of one with following signal decay (Fig. 6e, inset panel). This can be caused by the reflection of a US wave from the vessel’s wall providing an additional peak at the waveform.

**Figure 8:**
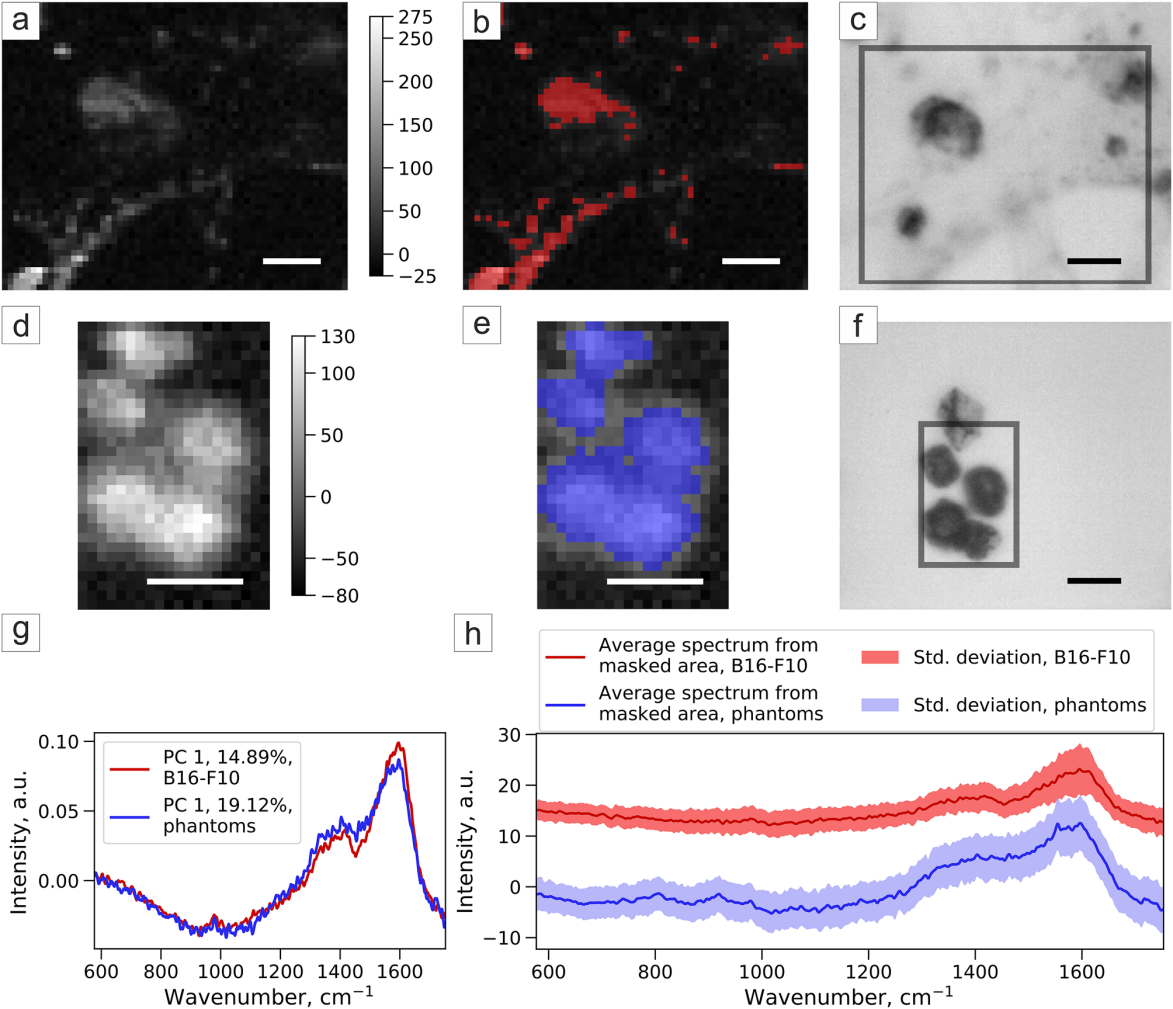
Principal component analysis of Raman spectroscopy of (**a**–**c**) B16F10 and (**d**–**f**) melanoma phantoms: (**a**,**d**) map of PCA component corresponding to melanin; (**b**,**e**) melanin-rich area selected by Otsu thresholding of PCA component map; (**c**,**f**) brightfield image, grey frame shows the scanning area; (**g**) Raman spectra of the component in PCA corresponding to melanin and (**h**) average Raman spectra with a standard deviation of masked areas of B16F10 and melanoma phantom samples.

Melanoma cells and melanoma cell phantoms were studied by Raman spectroscopy to ensure their similarity and the ability to use phantoms for further Raman system calibration (Fig. 8c,f). Principal component analysis (PCA) method was used to process the obtained Raman maps (Fig. 8a,d), melanin-rich area was selected by Otsu thresholding of the PCA component map (Fig. 8b,e). The component spectra (Fig. 8g) and the average Raman spectra from the masked area (Fig. 8h) are similar for the cells and phantoms and correspond to the same spectrum of melanin (Fig. S5, Supplementary).

## Discussion

In this study we developed the dynamic phantom of artificial blood flow. It represents different cell types, which, in combination with scattering-absorbing medium and a plastic tube, were successfully used to test high-speed signal processing in PAFC, aimed at the detection of fast-moving cells. Previous PA phantoms were static or contained slowly moving objects, and their speed was significantly slower compared with the blood flow velocity: 0.1 cm/s^19^ against the broad range from 0.3–5 mm/s in micro vessels to 20–50 cm/s in large blood vessels^42^, respectively. Moreover, different types of absorbing blood cells were modeled by one type of particles only, usually the magnetic ones, which were ranging in different sizes. Therefore, they did not simulate the blood flow properly, requiring a more adequate standardized model.

Here, novel hollow polymer and silica capsules of different sizes were produced to simulate blood cells and melanoma markers. The novel kind of phantom resembles blood both in its dynamic and optical properties, thus providing a well-defined model to calibrate flow cytometry systems with different detection modalities: PA, Raman, and potentially other imaging. The summary of cell and phantom characteristics is presented below (Table 1). The similarity of melanoma cell phantom absorbance spectrum and the spectrum of CTCs was shown to have a 0.6% difference, assessed by the cosine distance method. RBC phantoms spectrum possessed 540 and ~570 nm hemoglobin peaks, characteristic of natural RBCs. Due to the incorporation of the natural active substances into phantoms’ structure, their optical spectra resembled the same from natural cells providing high PA peaks from melanoma phantoms and forming background signals from RBC phantoms on the PA trace similar to the in vivo PAFC. The heterogeneity of melanoma phantom PA signals correlated with the same behavior for B16F10 cells, since their pigmentation was found to differ significantly from one cell to another.

**Table 1:** The main characteristics of real cells and their phantoms.

Target type	Target compartment	Material	Shape	Diameter, µm	Average melanin content, pg/object	Coefficient of absorption curve slope	Absorption at 980 nm wavelength, a.u.
B16F10 cells	Natural melanin	–	Spherical in an unattached state (circulating in blood)	13.4 ± 4.3	2.5 ± 0.5^46^	− 0.0031	0.95
Melanoma phantoms	Melanin from *Sepia Officinalis*	Polymers	Spherical	12 ± 3	12.6 ± 0.3	− 0.0027	1.05
	Target compartment	Material	Shape	Diameter, µm	Height of absorption peak at 540 nm, a.u.	Height of absorption peak at 570 nm, a.u.	Average RMS amplitude of PA signal, a.u.
RBCs	Hemoglobin	-	Spherical	6.9 ± 0.6	0.035	0.044	0.35–0.55
RBC phantom	Hemoglobin	Silica	Spherical	3.07 ± 0.76	0.043	0.038	0.138
	Target compartment	Material	Shape	Diameter, µm	–	–	–
WBCs	–	–	Spherical	7.6 ± 0.5	–	–	–
WBC phantom	–	Silica	Spherical	5.4 ± 0.6	–	–	–
	Target compartment	Material	Shape	Diameter, µm	–	–	–
Platelets	–	–	Spherical	1.7 ± 0.3	–	–	–
Platelet phantom	–	Silica	Spherical	1.1 ± 0.3	–	–	–

The additional feature of the developed system constitutes its alterability depending on the current needs and requirements. Besides, melanoma phantoms have the same rheological and PA properties as melanoma cells in whole blood, moreover, we can change concentration of phantom cells in the blood sample and, thus, to control the frequency of PA signals. Since the developed synthesis protocol does not limit the number of synthesized phantoms, we can make a sample with their high concentration for the calibration. It is advantageous in comparison to the patient’s blood, where the CTC’s appearance frequency is extremely low and allows us to check PAFC system’s detection limits. However, we can make a blood sample with a low number of phantoms to model the real patient’s blood.

Moreover, when compared to blood, the phantom has reproducibility and a significantly longer lifetime with proper handling and usage. The developed phantom allows us to compare results obtained by different users on different PAFC devices, provide metrological traceability of results, and regular calibration and maintenance of PAFC device performance. Current phantoms-related issued dealing with their slightly smaller size in comparison to natural cells and the issue of certain heterogeneity can be solved in the course of the following investigations. Moreover, by filling the capsules with substances providing different optical properties, the novel phantom can be used to calibrate devices using fluorescent, Raman, and other detection methods.

## Methods

### Materials

Melanin from *Sepia officinalis*, calcium chloride dihydrate, anhydrous sodium carbonate, poly(allylamine hydrochloride) (PAH, average M_W_ 70,000), poly(sodium 4-styrenesulfonate) (PSS, average M_W_ 70,000), sodium chloride, ethylenediaminetetraacetic acid disodium salt dihydrate (EDTA), poly(vinylpyrrolidone) (PVP, average M_W_ ~ 40,000), tetraethylorthosilicate (TEOS, ≥ 99.0%), Dulbecco’s modified Eagle medium (DMEM) with high glucose concentration were obtained from Sigma-Aldrich (USA). Dulbecco’s phosphate-buffered saline (DPBS), fetal bovine serum (FBS), penicillin–streptomycin, and 0.25% trypsin solution with 0.02% EDTA were obtained from Thermo Fisher Scientific (USA). Ammonia hydrous (analytic grade) was obtained from Sigma Tech (Russia). Isopropyl alcohol (chemically pure) was obtained from Reachim (Russia). All chemicals were used as received without further purification. Deionized water (DI water, specific resistivity higher than 18.2 MΩ·cm) from Milli-Q Direct 8 (Millipore, Merck KGaA, Darmstadt, Germany) water purification system was used to prepare all solutions.

The mouse's melanoma cell line (B16F10) was provided by the Department of Cell Engineering, Education and Research Institute of Nanostructures and Biosystems, Saratov State University, Saratov, Russia.

### The PAFC system description

The PAFC system consisted of a flow module, a nanosecond pulse laser with a light sheet optical scheme, a US detection system (Fig. 2), and a digital acquisition module. The flow system design includes the silicon capillary (SC, inner diameter: 300 µm) and motorized syringe pump (with a typical flow rate of 0.8 mm/s). The light sheet optical scheme is based on ytterbium fiber laser (YLPP-1-150V-30, IPG Photonics Corp.), cylindrical lens (CL, focal length 200 mm, LJ1653L1-B-N-BK7, Thorlabs Inc.), neutral density filters (diameter 25 mm, absorptive ND filters NE03B (ND = 0.3), NE10B (ND = 1.0), Thorlabs Inc.), and objective lens (MO, 8 ×, NA = 0.2, Lomo) in Galilean configuration. Geometrical sizes of the beam in the focal region at full width at half maximum (FWHM) are 5 × 360 × 30 µm for the width, length, and depth, respectively forming a light-sheet across the capillary and nearly diffraction-limited focusing along its axis. The pulse laser works at 1064 nm wavelength with a pulse repetition rate of 2 kHz and 2 ns pulse width. The portion of light was directed through a wedge beam splitter (BS) to the fast avalanche photodiode (APD, LFD-2A, USSR) with digital delay generator (DG 645, Stanford Research Systems, Inc.) for acquisition triggering of the ADC board. Pyroelectric Energy Sensor (PE 10-C, Vega, OPHIR) was used to measure pulse energy. Measured pulse energy after passing the objective lens was 70% of energy at the laser output. All energy levels on graphs correspond to nominal laser energy, i.e. 100 µJ pulse energy on the graph corresponds to 70 µJ of energy that reached the tube.

The ultrasonic system consisted of the custom US transducer (3.5 MHz single element transducer, Imasonic) with a preamplifier (5682, Olympus) and US coupling gel (MEDIAGEL, Geltek-Medica, Ltd). AlazarTech ATS-9350 board (12 bit, 500MS/s, AlazarTech) was used for digital signal acquisition.

### Melanoma cell phantom synthesis

CaCO_3_ particles were synthesized using chemical coprecipitation method^43^. To obtain the templates around 10 µm in diameter, 12 mL of 0.2 M CaCl_2_ and 3 mL of 1 M Na_2_CO_3_ solutions were mixed at 100 rpm by a magnetic stirrer for 35 s. Mixing was stopped, and the solution was left for 9 min to let particles form large raspberry-shaped crystallites. After that the precipitate was washed 3 times with deionized water and was ready for further application.

The suspension of nano- and microparticles of melanin from *Sepia officinalis* was used for loading into calcium carbonate microparticles (Fig. S2a,b). First, to make the average size of melanin particles less than several hundred nm to provide their effective embedding into the calcium carbonate pores, 10 mg/mL melanin suspension was treated by pulsed US (Bandelin Sonopuls HD 2070, 20 kHz, 70 W) in the course of 3 cycles (60% power, 3-min-long each). 0.15 M sodium chloride solution of PSS (0.05 mg/mL concentration) was added 2:1 by volume prior to US to stabilize the nano- and submicron particles obtained during treatment. Next, the nanoparticles were centrifuged for 5 min at 20,000*g* and washed two times with water. The concentration of the obtained suspension was measured relative to the calibration curve for pure melanin suspension based on melanin spectra measurements (Fig. S2c, Supplementary).

Melanin-contained CaCO_3_ templates were made by freezing-induced loading method introduced in^44^. 10 mg of microparticles were resuspended in 1 mL of prepared melanin aqueous solution (concentration 0.25 mg/mL), and then frozen under constant mixing to prevent aggregation. After 4 loading cycles, the average mass content of melanin in calcium carbonate was 7.59% ± 0.19% (Fig. S2d; Table S1).

To stabilize the particles and prevent them from recrystallization, the polymeric shell was formed by Layer-by-Layer assembly according to^45^. Poly(allylamine hydrochloride) (PAH) and poly(sodium 4-styrenesulfonate) (PSS) were chosen for the shell formation. 1 mL of each polymer 0.15 M NaCl solution in 1 mg/mL concentration was consequently added to phantoms suspension with subsequent mixing and washing steps. Thus, (PAH/PSS)_4_ polymeric shell was assembled. Finally, to prevent fast sedimentation, carbonate cores were dissolved by adding 2 mL of 0.2 M ethylenediaminetetraacetic acid (EDTA) with further mixing. The average diameter of synthesized phantoms was 12 ± 3 µm, measured with the ImageJ program from calibrated brightfield optical microscopy images. The spectrum of phantoms possesses a curved slope characteristic for melanin (Fig. 2c).

### RBC phantom

For RBC phantoms, the chemical precipitation method^26,27^ was used to create hemoglobin-contained spherical microparticles. 5 mL of 0.15 M CaCl_2_ and 5 mL of 0.15 M Na_2_CO_3_ were constantly stirring for 30 s at 400 rpm. For hemoglobin (Reachim, Russia) encapsulation, pure hemoglobin solution was added to CaCl_2_ until the concentration of 10 mg/mL was reached prior to stirring, and the equal volume of water was added to Na_2_CO_3_ solution. Then, the precipitated solution was incubated for 30 s, washed 2 times with deionized water, 1 time with ethanol, and dried at 50 °C. After synthesis, we made the silica shell on the phantoms, which prevents recrystallization process and, thus, prevents the release of hemoglobin molecules into solution. First, 10 mg of CaCO_3_ particles were mixed with 1 mL of poly(vinylpyrrolidone) (1 mg/mL) for 5 min and treated in the US bath after the addition of 0.5 mL ethanol. Then the suspension was added to 5 mL of isopropanol + 250 µL of 25% ammonia solution. For SiO_2_ formation 15 µL of TEOS was added, and the suspension was left mixing for 20 h. Then the particles were washed 2 times with isopropanol, and 2 times with water, and the cores were dissolved by 0.05 M EDTA water solution with the following washing steps.

### White blood cell phantom

The templates for silica spheres formation were made from calcium carbonate by chemical precipitation. We used the same method as for the CTC phantom, but at 120 rpm speed of magnetic rotor stirring. Then the CaCO_3_ cores were covered with silica (SiO_2_) shell using tetraethylorthosilicate (TEOS) polycondensation reaction. First, CaCO_3_ particles were mixed with 1 mL of poly(vinylpyrrolidone) (1 mg/mL), then the suspension was added to 9 mL of isopropanol + 250 µL of 25% ammonia solution. For SiO_2_ formation 5 µL of TEOS was added 3 times every 30 min, and the suspension was left mixing for 20 h. After that the particles were washed 2 times with isopropanol, and 2 times with water, and the cores were dissolved by 0.05 M EDTA water solution with the following washing steps. Resulting WBC phantoms have an average size of 5.4 µm (Fig. 4a,c).

### Platelets phantom

The platelet phantoms were made by silica spheres formation on 1 µm CaCO_3_ templates using the same protocol as for WBC phantoms formation. Calcium carbonate synthesis was carried out by chemical precipitation in ethylene glycol at 400 rpm stirring for 2 h. Then the precipitate was washed with water 3 times, and the particles were ready for further use. After the SiO_2_ shell formation, the cores were dissolved by EDTA. The average size of platelet phantoms was 1.08 µm. SEM images of phantoms are presented in Fig. 4b.

### Cell preparation

B16F10 cell culture was grown in the Dulbecco’s modified Eagle medium (DMEM) with high glucose concentration (Sigma-Aldrich, US) supplemented with 10% of the fetal bovine serum (FBS) and 1% of the penicillin–streptomycin at 37 °C in 5% CO_2_ and a humidified atmosphere. The culture medium was changed every three days until monolayer formation was observed. After monolayer formation, cells were detached by 0.25% trypsin solution with 0.02% EDTA. One part of the received cell suspension was mixed with 2 parts of the fresh cell culture medium for trypsin effect neutralization and centrifuged at 1000 rpm for 5 min. After that, the supernatant was aspirated, and the cell pellet was resuspended in an appropriate volume of DPBS. Finally, the cell suspension was stained by 0.4% Trypan blue solution (Invitrogen, US) for 5 min and counted by automated cell counter "Countess" (Invitrogen, US) to estimate cell viability and concentration of cells.

To obtain the fluorescent CLSM image of adherent B16F10 cells (Fig. 5a), they were pre-stained by Calcein AM, Mitotracker, and DAPI dyes, subsequently. First, Calcein AM was added 1:1000 to culture media to stain the membrane of living cells and left for 30 min. Then, cells were washed by DPBS, and 200 nM Mitotracker solution in culture media was added to adherent cells to stain mitochondria. After 15 min incubation and the same washing step, 1:1000 DAPI:culture media solution was added to B16F10 to stain cell nuclei and left for 30 min with the following washing.

For Raman spectroscopy (Fig. 8) cells were plated on a 35-mm Petri dish with a glass bottom covered by poly-l-lysine. The growth media was replaced by DPBS prior to the measurement to exclude media components’ peaks from the resulted spectrum.

### In vivo experiment

Laboratory animals were kept according to the instructions of the Saratov State Medical University (No. 82 dated January 30, 2020). The care protocol does not contradict the international principles of biomedical animal studies of the 1985 Geneva Convention. The mice from the Saratov State Medical University were studied under active research protocol approved by the Ethics Committee of Saratov State Medical University (wide approval No. 5) on 29.12.2018. The BALb/c mouse was selected for the experiment. It was 6–8 weeks old and weighing 20–25 g. The surgical part was performed using general anesthesia, intraperitoneal injection of drugs [a mixture of Zoletil (40 mg per kg, 50 μL, Virbac SA, Carros, France), and 2% Rometar (10 μL and 10 mg per kg, Spofa, Czech Republic)]. At the end of the experiment, the animal was sacrificed with an overdose of anesthesia.

The mouse carotid artery was cannulated, and 10^6^ of B16F10 cells in DPBS buffer were injected using syringe pump (AL-1000, World Precision Instruments, USA). The volume of the fluid coming out of the syringe during minute was 40 μL. The PA signals were detected on the femoral artery using US transducer. Successful alignment on the vessel was characterized by the maximum of acoustic response, as well as the obvious pulsation of the arterial vessel.

### Microscopy methods

#### Electronic microscopy

Phantom samples were visualized with SEM [MIRA II LMU device (Tescan, Czech Republic)] at an operating voltage of 30 kV. To provide the SEM measurements, 10 μL of water particle suspension was placed on a silicon substrate and dried overnight. The surface of the sample was then covered with gold before SEM images were taken.

#### Convectional optical microscopy

Optical and fluorescent images of cells and phantoms were obtained using optical inverted microscope Olympus IX73 with Olympus DP73 camera and objective UPlanFl 60 ×/1.25 Oil Iris with immersion objective lens. The phase-contrast mode was used to visualize properly transparent objects, in particular, the shell of phantoms and the low optical density of cells, lacking high-absorbing inclusions.

#### Confocal laser scanning microscopy

CLSM images of cells were obtained with the Leica TCS SP8 X system using 20 ×/0.7 n.a. objective lens.

#### Raman spectroscopy

Raman maps were obtained with the Renishaw inVia system using 532-nm laser focused through 50 ×/0.5 n.a. objective. For melanoma phantom measurements, laser power of 0.3 mW and exposure time of 15 s were used. B16F10 cells were measured using 0.15 mW laser power and 1 s exposure time. Raman data were recorded using WiRE software v.4.4.

### Human blood experiments

The PA measurements were processed in vitro with venous blood collected into a heparinized tube from a healthy human volunteer (one of the authors). The respondent was a 22-year-old Caucasian woman. All experiments were performed in accordance with the relevant guidelines and regulations at Saratov State University and informed consent was obtained prior to blood collection, under a research protocol approved by the Ethics Committee of Saratov State Medical University (wide approval No. 6) on 06.02.2018.

### Data processing

All the PA measurements were performed at least three times with different samples to characterize the repeatability of the measurements. PDMS showed no significant change in the generated PA signal amplitudes The PA signals were acquired, and points at least 3-sigma above the blood background were considered as signals from high-absorbing objects. PA signal rate is described as a number of such signals per minute. Collected data (mean values) are presented as mean ± standard deviation. PCA of Raman maps was compiled with a scikit-learn library, statistical analysis was done with the Numpy library of Python v.3.6 and the Jupyter Notebook software.

## Supplementary Information

Supplementary Video.

Supplementary Information.

## References

[1] Wang LV, Hu S (2012). Photoacoustic tomography: In vivo imaging from organelles to organs. Science.

[2] Hekman MCH (2018). Positron emission tomography/computed tomography with 89Zr-girentuximab can aid in diagnostic dilemmas of clear cell renal cell carcinoma suspicion. Eur. Urol..

[3] Chakravarty P, Qian W, El-Sayed MA, Prausnitz MR (2010). Delivery of molecules into cells using carbon nanoparticles activated by femtosecond laser pulses. Nat. Nanotechnol..

[4] Galanzha EI, Shashkov EV, Spring PM, Suen JY, Zharov VP (2009). In vivo, noninvasive, label-free detection and eradication of circulating metastatic melanoma cells using two-color photoacoustic flow cytometry with a diode laser. Cancer Res..

[5] Galanzha EI, Zharov VP (2012). Photoacoustic flow cytometry. Methods.

[6] Galanzha EI (2009). In vivo magnetic enrichment and multiplex photoacoustic detection of circulating tumour cells. Nat. Nanotechnol..

[7] O’Donnell M (2013). Can molecular imaging enable personalized diagnostics? An example using magnetomotive photoacoustic imaging. Ann. Biomed. Eng..

[8] Mallidi S, Luke GP, Emelianov S (2011). Photoacoustic imaging in cancer detection, diagnosis, and treatment guidance. Trends Biotechnol..

[9] Razansky D (2009). Multispectral opto-acoustic tomography of deep-seated fluorescent proteins in vivo. Nat. Photonics.

[10] Wang J, Jokerst JV (2016). Stem cell imaging: Tools to improve cell delivery and viability. Stem Cells Int..

[11] Galanzha EI (2019). In vivo liquid biopsy using Cytophone platform for photoacoustic detection of circulating tumor cells in patients with melanoma. Sci. Transl. Med..

[12] Cai C (2016). In vivo photoacoustic flow cytometry for early malaria diagnosis. Cytom. Part A.

[13] Nolan J (2016). In vivo flow cytometry of circulating tumor-associated exosomes. Anal. Cell. Pathol..

[14] Karpiouk AB (2008). Combined ultrasound and photoacoustic imaging to detect and stage deep vein thrombosis: Phantom and ex vivo studies. J. Biomed. Opt..

[15] Tuchin VV, Tárnok A, Zharov VP (2011). In vivo flow cytometry: A horizon of opportunities. Cytom. Part A.

[16] Avigo C (2015). Organosilicon phantom for photoacoustic imaging. J. Biomed. Opt..

[17] Vogt WC, Jia C, Wear KA, Garra BS, Joshua Pfefer T (2016). Biologically relevant photoacoustic imaging phantoms with tunable optical and acoustic properties. J. Biomed. Opt..

[18] Jiang Y, Forbrich A, Harrison T, Zemp RJ (2012). Blood oxygen flux estimation with a combined photoacoustic and high-frequency ultrasound microscopy system: A phantom study. J. Biomed. Opt..

[19] Thatcher JE (2014). Dynamic tissue phantoms and their use in assessment of a noninvasive optical plethysmography imaging device. SPIE Proc. Smart Biomed. Physiol. Sens. Technol. XI.

[20] Quan KM, Christison GB, MacKenzie HA, Hodgson P (1993). Glucose determination by a pulsed photoacoustic technique: An experimental study using a gelatin-based tissue phantom. Phys. Med. Biol..

[21] Hoelen CG, Pongers R, Hamhuis G, de Mul FFM, Greve J (1998). Photoacoustic blood cell detection and imaging of blood vessels in phantom tissue. SPIE Proc. Opt. Imaging Tech. Biomonit. III.

[22] Jawad HJ, Sarimollaoglu M, Biris AS, Zharov VP (2018). Dynamic blood flow phantom with negative and positive photoacoustic contrasts. Biomed. Opt. Express.

[23] Juratli MA (2015). In vivo long-term monitoring of circulating tumor cells fluctuation during medical interventions. PLoS ONE.

[24] Koonce NA (2017). Real-time monitoring of circulating tumor cell (CTC) release after nanodrug or tumor radiotherapy using in vivo flow cytometry. Biochem. Biophys. Res. Commun..

[25] Galanzha EI (2016). In vivo acoustic and photoacoustic focusing of circulating cells. Sci. Rep..

[26] Volodkin DV, Petrov AI, Prevot M, Sukhorukov GB (2004). Matrix polyelectrolyte microcapsules: New system for macromolecule encapsulation. Langmuir.

[27] Xiong Y (2012). Hemoglobin-based oxygen carrier microparticles: Synthesis, properties, and in vitro and in vivo investigations. Biomacromol.

[28] Xiong Y (2013). Nonvasoconstrictive hemoglobin particles as oxygen carriers. ACS Nano.

[29] Meinke M, Müller G, Helfmann J, Friebel M (2007). Optical properties of platelets and blood plasma and their influence on the optical behavior of whole blood in the visible to near infrared wavelength range. J. Biomed. Opt..

[30] Gómez-Cuadrado L, Tracey N, Ma R, Qian B, Brunton VG (2017). Mouse models of metastasis: Progress and prospects. Dis. Model. Mech..

[31] Rizzitelli S (2015). Sonosensitive theranostic liposomes for preclinical in vivo MRI-guided visualization of doxorubicin release stimulated by pulsed low intensity non-focused ultrasound. J. Control. Release.

[32] Rodriguez-Blanco JD, Shaw S, Benning LG (2011). The kinetics and mechanisms of amorphous calcium carbonate (ACC) crystallization to calcite, viavaterite. Nanoscale.

[33] Gebauer D, Völkel A, Cölfen H (2008). Stable prenucleation calcium carbonate clusters. Science.

[34] Lei M, Li PG, Sun ZB, Tang WH (2006). Effects of organic additives on the morphology of calcium carbonate particles in the presence of CTAB. Mater. Lett..

[35] Wang L, Sondi I, Matijević E (1999). Preparation of uniform needle-like aragonite particles by homogeneous precipitation. J. Colloid Interface Sci..

[36] Takiguchi M, Igarashi K, Azuma M, Ooshima H (2006). Flowerlike agglomerates of calcium carbonate crystals formed on an eggshell membrane. Cryst. Growth Des..

[37] Cölfen H (2003). Precipitation of carbonates: Recent progress in controlled production of complex shapes. Curr. Opin. Colloid Interface Sci..

[38] Ouhenia S, Chateigner D, Belkhir MA, Guilmeau E, Krauss C (2008). Synthesis of calcium carbonate polymorphs in the presence of polyacrylic acid. J. Cryst. Growth.

[39] Kumar GS, Thamizhavel A, Girija EK (2012). Microwave conversion of eggshells into flower-like hydroxyapatite nanostructure for biomedical applications. Mater. Lett..

[40] Han, J., Kamber, M. & Pei, J. Getting to know your data. in *Data Mining* 39–82 (Elsevier, 2012).

[41] Galanzha E, Zharov V (2013). Circulating tumor cell detection and capture by photoacoustic flow cytometry in vivo and ex vivo. Cancers.

[42] Zharov VP, Galanzha EI, Tuchin VV (2006). In vivo photothermal flow cytometry: Imaging and detection of individual cells in blood and lymph flow. J. Cell. Biochem..

[43] Sukhorukov GB (2004). Porous calcium carbonate microparticles as templates for encapsulation of bioactive compounds. J. Mater. Chem..

[44] German SV (2018). High-efficiency freezing-induced loading of inorganic nanoparticles and proteins into micron- and submicron-sized porous particles. Sci. Rep..

[45] Sukhorukov GB (1998). Stepwise polyelectrolyte assembly on particle surfaces: A novel approach to colloid design. Polym. Adv. Technol..

[46] Chung S, Lim GJ, Lee JY (2019). Quantitative analysis of melanin content in a three-dimensional melanoma cell culture. Sci. Rep..

