# Supplementary material for: Dynamic blood flow phantom for in vivo liquid biopsy standardization
Source: Sci Rep. 2021 Jan 13;11:1185. doi: 10.1038/s41598-020-80487-8 (PMC7806591; doi:10.1038/s41598-020-80487-8)
Supplement: Supplementary file 2 — Supplementary Information. [file 41598_2020_80487_MOESM2_ESM.pdf]

Supplementary information

Dynamic blood flow phantom for *in vivo* liquid biopsy standardization

Anastasiia Kozlova*, Daniil Bratashov, Oleg Grishin, Arkadii Abdurashitov, Ekaterina Prikhozhdenko, Roman Verkhovskii, Natalia Shushunova, Evgenii Shashkov, Vladimir P. Zharov*, Olga Inozemtseva

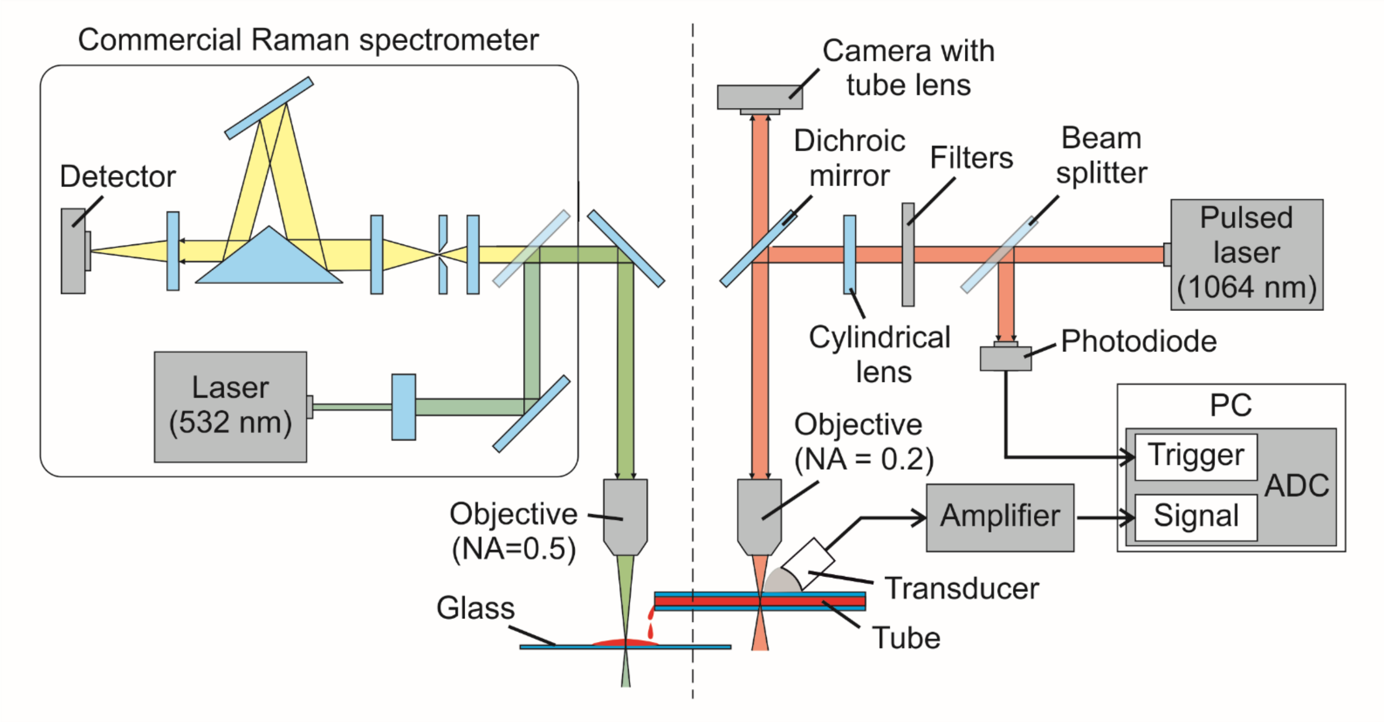

Figure S1. Scheme of the experiment, including Raman (left) and photoacoustic (right) setups. PC - personal computer; ADC - analog-to-digital converter.

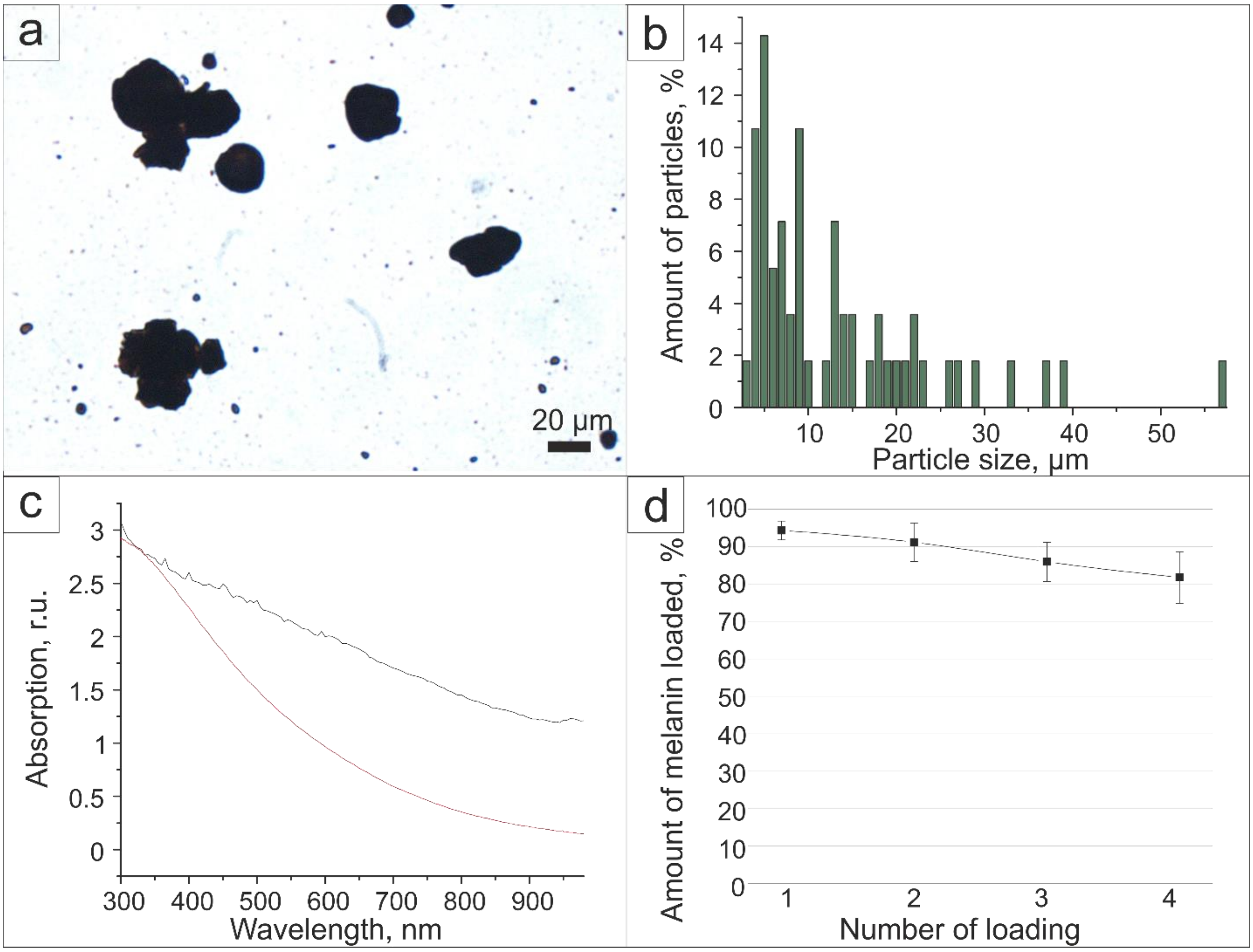

Figure S2. (**a**) Optical image of melanin microparticles in initial suspension, (**b**) melanin microparticles size distribution, (**c**) absorption spectra of melanin suspension (orange curve), and suspension of melanoma phantoms containing melanin (black curve), (**d**) amount of loaded melanin vs. a number of loading cycles.

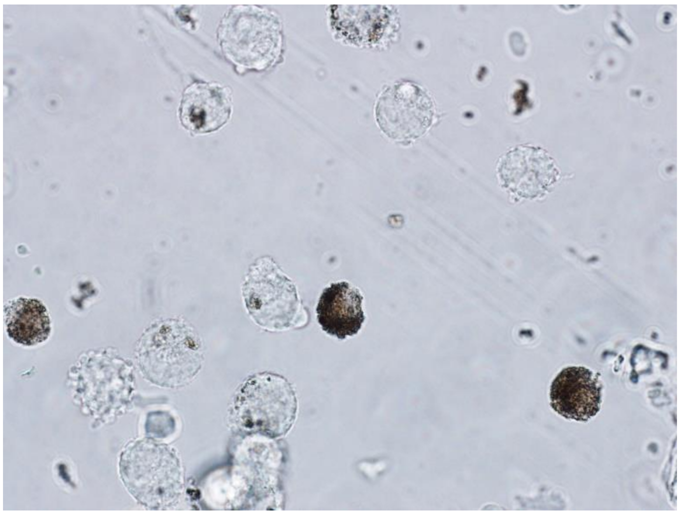

Figure S3. Optical microscopy image of detached B16F10 cells.

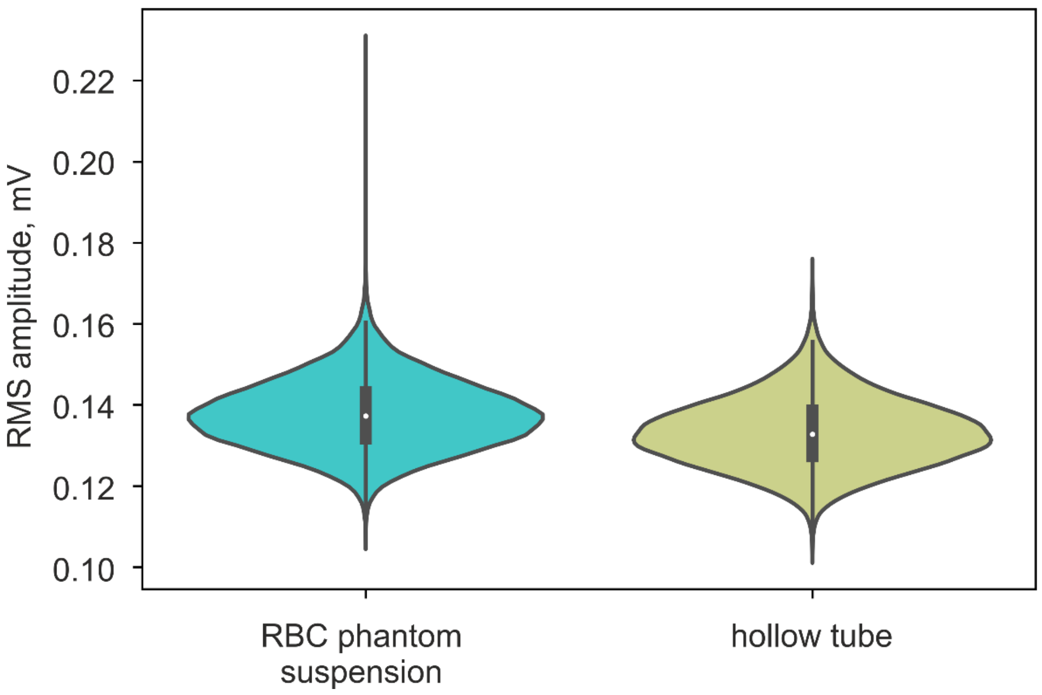

Figure S4. PA signal background. The mean value from the PA signals of the RBC phantom suspension and hollow tube.

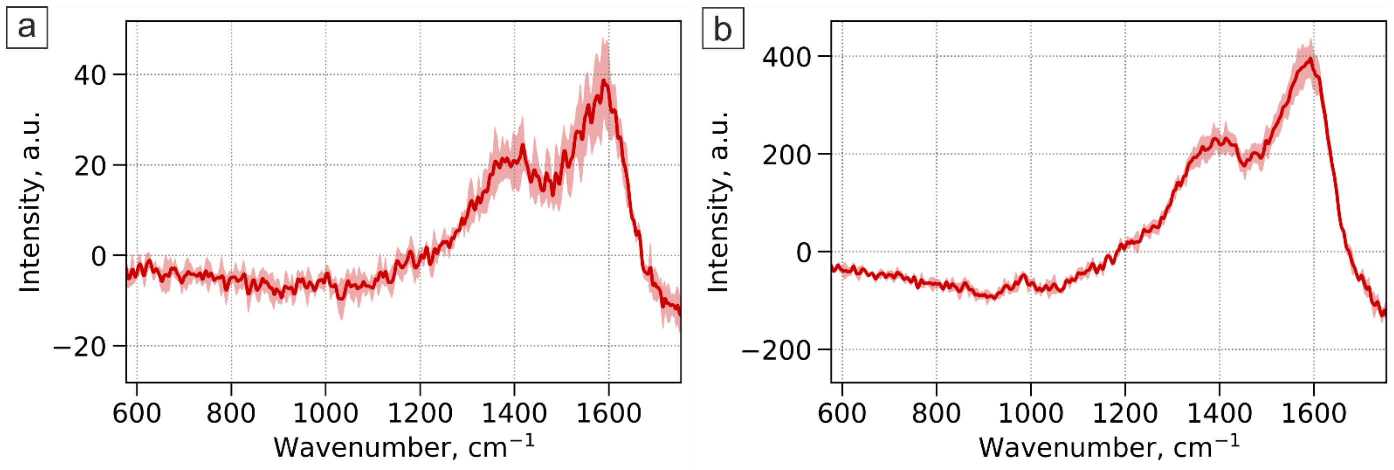

Figure S5. Average Raman spectra with a standard deviation of (**a**) dry and (**b**) wet melanin microparticles.

Table S1 – Statistics of melanin loading to phantoms

Number of loading	№1, mg	№2, mg	№3, mg	№4, mg	Summary, mg	Loading per 1 mg $CaCO_3$, mg	Phantoms amount	Loading per 1 phantom, pg
1	0.238	0.194	0.179	0.177	0.78	0.078	$6{\cdot}10^7$	13
	95.65%	89.01%	76.97%	81.71%	7.80%			
2	0.191	0.2	0.19	0.186	0.76	0.076	$6{\cdot}10^7$	12.7
	93.32%	91.42%	90.76%	86.69%	7.63%			
3	0.19	0.203	0.181	0.186	0.758	0.076	$6{\cdot}10^7$	12.6
	92.67%	93.05%	86.61%	86.61%	7.58%			
4	0.213	0.192	0.174	0.155	0.735	0.073	$6{\cdot}10^7$	12.2
	90.66%	82.09%	82.95%	72.03%	7.35%			

Video S1. Laser irradiation of absorbing objects in the flow following by bubble formation.
